# Supplementary material for: Genetic diversity and structure of the critically endangered Artocarpus annulatus, a crop wild relative of jackfruit (A. heterophyllus)
Source: PeerJ. 2020 Sep 21;8:e9897. doi: 10.7717/peerj.9897 (PMC7513743; doi:10.7717/peerj.9897)
Supplement: Table S1 — Latitude and longitude are not reported due to critical endangered status. All associated data were not always collected for all samples, resulting in some incomplete data. This was due to the very rough terrain, making it difficult to access trunks for exact DBH (diameter at breast height) measurements. Tree heights are estimates. For the purposes of differentiating age, any sample less than 1 meter tall was considered a seedling; any sample taller than 1 meter and with a dbh less than 5.5 cm was considered a sapling; and any sample with a dbh of 5.5 cm or more was considered mature due to the observation that trees of that size often had signs of past reproduction. All other samples were considered saplings. Collection numbers in bold have voucher specimens deposited in SAR. For all other samples, only dried leaf material was collected. [file peerj-08-9897-s002.docx]

**Supplementary Information**

**Table S1** Sample information from all 131 *A. annulatus* samples. Latitude and longitude are not reported due to critical endangered status. All associated data were not always collected for all samples, resulting in some incomplete data. This was due to the very rough terrain, making it difficult to access trunks for exact DBH (diameter at breast height) measurements. Tree heights are estimates. For the purposes of differentiating age, any sample less than 1 meter tall was considered a seedling; any sample taller than 1 meter and with a dbh less than 5.5 cm was considered a sapling; and any sample with a dbh of 5.5 cm or more was considered mature due to the observation that trees of that size often had signs of past reproduction. All other samples were considered saplings. Collection numbers in bold have voucher specimens deposited in SAR. For all other samples, only dried leaf material was collected.

| **Collector** | **Collector Number** | **Collection Date** | **Locality description** | **DBH (cm)** | **Height (m)** | **Age class** |
| --- | --- | --- | --- | --- | --- | --- |
| E. Gardner | **601** | 17-Jul-2017 | G. Gayu | 10 | 12 | Mature |
| E. Gardner | **602** | 17-Jul-2017 | G. Gayu | 4 | 6 | Sapling |
| E. Gardner | 603 | 17-Jul-2017 | G. Gayu |  | 2 | Sapling |
| E. Gardner | 604 | 17-Jul-2017 | G. Gayu |  | 3 | Sapling |
| E. Gardner | 605 | 17-Jul-2017 | G. Gayu | 3 | 5 | Sapling |
| E. Gardner | 606 | 17-Jul-2017 | G. Gayu |  | 1 | Sapling |
| E. Gardner | 607 | 17-Jul-2017 | G. Gayu |  | 1.5 | Sapling |
| E. Gardner | 608 | 17-Jul-2017 | G. Gayu |  | 1.5 | Sapling |
| E. Gardner | 609 | 17-Jul-2017 | G. Gayu |  | 1.5 | Sapling |
| E. Gardner | 610 | 17-Jul-2017 | G. Gayu |  | 1.5 | Sapling |
| E. Gardner | 611 | 17-Jul-2017 | G. Gayu |  | 1.5 | Sapling |
| E. Gardner | 612 | 17-Jul-2017 | G. Gayu |  | 1 | Sapling |
| E. Gardner | **613** | 17-Jul-2017 | G. Gayu | 3 | 8 | Sapling |
| E. Gardner | 614 | 17-Jul-2017 | G. Gayu | 3 | 2.5 | Sapling |
| E. Gardner | **615** | 17-Jul-2017 | G. Gayu | 3 | 8 | Sapling |
| E. Gardner | **616** | 17-Jul-2017 | G. Gayu | 8 | 10 | Mature |
| E. Gardner | **617** | 17-Jul-2017 | G. Gayu | 16 | 12 | Mature |
| E. Gardner | 618 | 17-Jul-2017 | G. Gayu | 12 | 12 | Mature |
| E. Gardner | 619 | 17-Jul-2017 | G. Gayu |  | 3 | Sapling |
| E. Gardner | **620** | 17-Jul-2017 | G. Gayu | 15 | 10 | Mature |
| E. Gardner | 621 | 17-Jul-2017 | G. Gayu |  | 1 | Sapling |
| E. Gardner | 622 | 17-Jul-2017 | G. Gayu |  | 0.5 | Seedling |
| E. Gardner | **639** | 19-Jul-2017 | G. Bedoh | 6 | 6 | Mature |
| E. Gardner | 640 | 19-Jul-2017 | G. Bedoh | 10 | 7 | Mature |
| E. Gardner | 641 | 19-Jul-2017 | G. Bedoh |  | 1 | Sapling |
| E. Gardner | 642 | 19-Jul-2017 | G. Bedoh |  | 2 | Sapling |
| E. Gardner | 643 | 19-Jul-2017 | G. Bedoh |  | 0.5 | Seeding |
| E. Gardner | 644 | 19-Jul-2017 | G. Bedoh |  | 0.3 | Seedling |
| E. Gardner | 645 | 19-Jul-2017 | G. Bedoh | 8 | 8 | Mature |
| E. Gardner | 646 | 19-Jul-2017 | G. Bedoh | 3 | 6 | Sapling |
| E. Gardner | 647 | 19-Jul-2017 | G. Bedoh | 5 | 4 | Sapling |
| E. Gardner | 648 | 19-Jul-2017 | G. Bedoh | 12 | 9 | Mature |
| E. Gardner | 649 | 19-Jul-2017 | G. Bedoh | 2 | 1 | Sapling |
| E. Gardner | **650** | 19-Jul-2017 | G. Bedoh | 5 | 8 | Sapling |
| E. Gardner | 651 | 19-Jul-2017 | G. Bedoh | 9 | 10 | Mature |
| E. Gardner | 652 | 19-Jul-2017 | G. Bedoh | 6 | 6 | Mature |
| E. Gardner | 653 | 19-Jul-2017 | G. Bedoh | 8 | 7 | Mature |
| E. Gardner | 654 | 19-Jul-2017 | G. Bedoh | 8 | 10 | Mature |
| E. Gardner | 655 | 19-Jul-2017 | G. Bedoh | 5 | 7 | Sapling |
| E. Gardner | 656 | 19-Jul-2017 | G. Bedoh |  | 1 | Sapling |
| E. Gardner | 657 | 19-Jul-2017 | G. Bedoh |  | 1 | Sapling |
| E. Gardner | 658 | 19-Jul-2017 | G. Bedoh |  | 1 | Sapling |
| E. Gardner | 659 | 19-Jul-2017 | G. Bedoh |  | 2 | Sapling |
| E. Gardner | 660 | 19-Jul-2017 | G. Bedoh |  | 2 | Sapling |
| E. Gardner | 661 | 19-Jul-2017 | G. Bedoh | 20 | 8 | Mature |
| E. Gardner | 662 | 19-Jul-2017 | G. Bedoh | 15 | 10 | Mature |
| E. Gardner | 663 | 19-Jul-2017 | G. Bedoh |  | 0.5 | Seedling |
| E. Gardner | 664 | 19-Jul-2017 | G. Bedoh | 21 | 12 | Mature |
| E. Gardner | 665 | 20-Jul-2017 | G. Manok |  | 2 | Sapling |
| E. Gardner | 666 | 20-Jul-2017 | G. Manok | 16 | 11 | Mature |
| E. Gardner | 667 | 20-Jul-2017 | G. Manok | 3 | 6 | Sapling |
| E. Gardner | 668 | 20-Jul-2017 | G. Manok | 4 | 7 | Sapling |
| E. Gardner | 669 | 20-Jul-2017 | G. Manok | 6 | 8 | Mature |
| E. Gardner | 670 | 20-Jul-2017 | G. Manok | 12 | 7 | Mature |
| E. Gardner | 671 | 20-Jul-2017 | G. Manok | 10 | 10 | Mature |
| E. Gardner | 672 | 20-Jul-2017 | G. Manok | 12 | 10 | Mature |
| E. Gardner | 673 | 20-Jul-2017 | G. Manok | 12 | 10 | Mature |
| E. Gardner | 674 | 20-Jul-2017 | G. Manok | 10 | 8 | Mature |
| E. Gardner | 675 | 20-Jul-2017 | G. Manok | 8 | 8 | Mature |
| E. Gardner | 676 | 20-Jul-2017 | G. Manok | 3 | 3 | Sapling |
| E. Gardner | 677 | 20-Jul-2017 | G. Manok | 20 | 11 | Mature |
| E. Gardner | 678 | 20-Jul-2017 | G. Manok | 8 | 8 | Mature |
| E. Gardner | 679 | 20-Jul-2017 | G. Manok | 10 | 6 | Mature |
| E. Gardner | 680 | 20-Jul-2017 | G. Manok | 3 | 4 | Sapling |
| E. Gardner | 681 | 20-Jul-2017 | G. Manok | 12 | 10 | Mature |
| E. Gardner | 682 | 20-Jul-2017 | G. Manok | 1.5 | 3 | Sapling |
| E. Gardner | 683 | 20-Jul-2017 | G. Manok | 10 | 12 | Mature |
| E. Gardner | 684 | 20-Jul-2017 | G. Manok | 3 | 7 | Sapling |
| E. Gardner | 685 | 20-Jul-2017 | G. Manok |  | 2 | Sapling |
| N. Zerega | **985** | 11-Jul-2016 | G. Mentawa | 15 | 8 | Mature |
| N. Zerega | 986 | 11-Jul-2016 | G. Mentawa |  | <1 | Seedling |
| N. Zerega | 987 | 11-Jul-2016 | G. Mentawa |  | <1 | Seedling |
| N. Zerega | 988 | 11-Jul-2016 | G. Mentawa |  | <1 | Seedling |
| N. Zerega | 989 | 11-Jul-2016 | G. Mentawa |  | <1 | Seedling |
| N. Zerega | 990 | 11-Jul-2016 | G. Mentawa |  | <1 | Seedling |
| N. Zerega | **991** | 11-Jul-2016 | G. Mentawa |  | 1 | Sapling |
| N. Zerega | 992 | 11-Jul-2016 | G. Mentawa | Split trunk: 8, 15 | 14 | Mature |
| N. Zerega | 993 | 11-Jul-2016 | G. Mentawa | 8 | 3 | Mature |
| N. Zerega | 994 | 11-Jul-2016 | G. Mentawa | 12 | 8 | Mature |
| N. Zerega | 995 | 11-Jul-2016 | G. Mentawa | 8 | 8 | Mature |
| N. Zerega | 996 | 11-Jul-2016 | G. Mentawa | 9 |  | Mature |
| N. Zerega | 997 | 11-Jul-2016 | G. Mentawa | 3 | 5 | Sapling |
| N. Zerega | 998 | 11-Jul-2016 | G. Mentawa | 1 | 1 | Sapling |
| N. Zerega | 1001 | 11-Jul-2016 | G. Mentawa |  | <1 | Seedling |
| N. Zerega | **1003** | 13-Jul-2016 | G. Teng Bukap | 2 | 2.5 | Sapling |
| N. Zerega | 1004 | 13-Jul-2016 | G. Teng Bukap | 1 | 2 | Sapling |
| N. Zerega | 1005 | 13-Jul-2016 | G. Teng Bukap | 5.5 | 9 | Mature |
| N. Zerega | 1006 | 13-Jul-2016 | G. Teng Bukap | 7 | 10 | Mature |
| N. Zerega | 1007 | 13-Jul-2016 | G. Teng Bukap | 0.5 | 2.5 | Sapling |
| N. Zerega | 1008 | 13-Jul-2016 | G. Teng Bukap |  | <1 | Seedling |
| N. Zerega | 1009 | 13-Jul-2016 | G. Teng Bukap |  | <1 | Seedling |
| N. Zerega | 1010 | 13-Jul-2016 | G. Teng Bukap |  | <1 | Seedling |
| N. Zerega | 1011 | 13-Jul-2016 | G. Teng Bukap |  | <1 | Seedling |
| N. Zerega | 1012 | 13-Jul-2016 | G. Teng Bukap |  | <1 | Seedling |
| N. Zerega | 1013 | 13-Jul-2016 | G. Teng Bukap |  | <1 | Seedling |
| N. Zerega | 1014 | 13-Jul-2016 | G. Teng Bukap | 2 | 4 | Sapling |
| N. Zerega | 1015 | 13-Jul-2016 | G. Teng Bukap | 4 | 5 | Sapling |
| N. Zerega | 1016 | 13-Jul-2016 | G. Teng Bukap |  | <1 | Seedling |
| N. Zerega | 1017 | 13-Jul-2016 | G. Teng Bukap |  | <1 | Seedling |
| N. Zerega | 1018 | 13-Jul-2016 | G. Teng Bukap | 0.5 | 1.5 | Sapling |
| N. Zerega | 1019 | 13-Jul-2016 | G. Teng Bukap |  | <1 | Seedling |
| N. Zerega | 1020 | 13-Jul-2016 | G. Teng Bukap |  | <1 | Seedling |
| N. Zerega | 1021 | 13-Jul-2016 | G. Teng Bukap | 2 | 4 | Sapling |
| N. Zerega | 1022 | 13-Jul-2016 | G. Teng Bukap |  | <1 | Seedling |
| N. Zerega | 1023 | 13-Jul-2016 | G. Teng Bukap |  | <1 | Seedling |
| N. Zerega | 1024 | 13-Jul-2016 | G. Teng Bukap |  | <1 | Seedling |
| N. Zerega | 1025 | 13-Jul-2016 | G. Teng Bukap |  | <1 | Seedling |
| N. Zerega | 1026 | 13-Jul-2016 | G. Teng Bukap |  | <1 | Seedling |
| N. Zerega | 1027 | 13-Jul-2016 | G. Teng Bukap |  | <1 | Seedling |
| N. Zerega | 1028 | 13-Jul-2016 | G. Teng Bukap | 0.5 | 1.5 | Sapling |
| N. Zerega | 1029 | 13-Jul-2016 | G. Teng Bukap | 10.4 | 14 | Mature |
| N. Zerega | 1030 | 13-Jul-2016 | G. Teng Bukap | 0.5 | 2.5 | Sapling |
| N. Zerega | 1031 | 13-Jul-2016 | G. Teng Bukap | 1.5 | 2.5 | Sapling |
| N. Zerega | 1032 | 13-Jul-2016 | G. Teng Bukap |  | 1 | Sapling |
| N. Zerega | 1033 | 13-Jul-2016 | G. Teng Bukap | 2 | 3.5 | Sapling |
| N. Zerega | 1034 | 13-Jul-2016 | G. Teng Bukap | 9.4 | 11 | Mature |
| N. Zerega | 1035 | 13-Jul-2016 | G. Teng Bukap |  | 2 | Sapling |
| N. Zerega | 1036 | 13-Jul-2016 | G. Teng Bukap |  | 2 | Sapling |
| N. Zerega | 1037 | 13-Jul-2016 | G. Teng Bukap |  | 1 | Sapling |
| N. Zerega | 1038 | 13-Jul-2016 | G. Teng Bukap |  | <1 | Seedling |
| N. Zerega | 1039 | 13-Jul-2016 | G. Teng Bukap |  | 1.5 | Sapling |
| N. Zerega | 1040 | 13-Jul-2016 | G. Teng Bukap |  | 0.5 | Seedling |
| N. Zerega | 1041 | 13-Jul-2016 | G. Teng Bukap |  | 0.5 | Seedling |
| N. Zerega | 1042 | 13-Jul-2016 | G. Teng Bukap | 3 | 4.5 | Sapling |
| N. Zerega | 1043 | 13-Jul-2016 | G. Teng Bukap | 10 | 12 | Mature |
| N. Zerega | 1044 | 13-Jul-2016 | G. Teng Bukap |  | <1 | Seedling |
| N. Zerega | 1045 | 13-Jul-2016 | G. Teng Bukap |  | 1 | Sapling |
| N. Zerega | 1046 | 13-Jul-2016 | G. Teng Bukap |  | 1 | Sapling |
| N. Zerega | 1047 | 13-Jul-2016 | G. Teng Bukap |  | 0.5 | Seedling |
| N. Zerega | 1048 | 13-Jul-2016 | G. Teng Bukap | 11 | 12 | Mature |
| N. Zerega | 1049 | 13-Jul-2016 | G. Teng Bukap | 1.8 | 4 | Sapling |
