# Supplementary material for: Genetic diversity and structure of the critically endangered Artocarpus annulatus, a crop wild relative of jackfruit (A. heterophyllus)
Source: PeerJ. 2020 Sep 21;8:e9897. doi: 10.7717/peerj.9897 (PMC7513743; doi:10.7717/peerj.9897)
Supplement: Table S2 — Microsatellite marker MAA178 amplified two regions and is notated as two loci, 178A and 178B. Loci in bold italics were excluded from further analysis due to being monomorphic (MAA122 and MAA140), functionally monomorphic (MAA54) or having evidence of null alleles (MAA105). [file peerj-08-9897-s003.docx]

**Supplementary Information**

**Table S2** Genetic diversity measures for ten microsatellite loci across all populations. Microsatellite marker MAA178 amplified two regions and is notated as two loci, 178A and 178B. Loci in bold italics were excluded from further analysis due to being monomorphic (MAA122 and MAA140), functionally monomorphic (MAA54) or having evidence of null alleles (MAA105).

| **Locus** | **N** | **N_a_** | **N_e_** | **H_o_** | **H_e_** | **F_is_** | **Null Allele Freq** |
| --- | --- | --- | --- | --- | --- | --- | --- |
| ***MAA122*** | ***82*** | ***1*** | ***1.00*** | ***-*** | ***-*** | ***-*** | ***-*** |
| MAA26 | 111 | 6 | 1.72 | 0.43 | 0.42 | -0.03 | -0.023 |
| ***MAA140*** | ***79*** | ***1*** | ***1.00*** | ***-*** | ***-*** | ***-*** | ***0*** |
| MAA156 | 121 | 5 | 1.45 | 0.39 | 0.31 | -0.25 | -0.113 |
| ***MAA105*** | ***93*** | ***5*** | ***1.30*** | ***0.18*** | ***0.22*** | ***-0.16*** | ***0.206*** |
| MAA182 | 93 | 2 | 1.01 | 0.08 | 0.09 | 0.18 | -0.060 |
| MAA178A | 103 | 6 | 2.63 | 0.52 | 0.62 | 0.15 | 0.098 |
| MAA178B | 119 | 8 | 2.19 | 0.61 | 0.54 | -0.13 | -0.004 |
| MAA196 | 123 | 6 | 2.07 | 0.69 | 0.52 | -0.34 | -0.136 |
| ***54*** | ***94*** | ***4*** | ***2.16*** | ***1.00*** | ***0.53*** | ***-0.88*** | ***-0.765*** |
| Mean | 101.8 | 2.48 | 1.62 | 0.44 | 0.31 | -0.27 | -0.080 |
| SE | 5.085 | 0.18 | 0.08 | 0.05 | 0.03 | 0.11 | 0.082 |

N = sample size, N_a_ = no. of different alleles, N_e_ = no. of effective alleles, H_o_ = observed heterozygosity, H_e_ = expected heterozygosity, F = Fixation Index [1 – (H_o_/H_e_)]
