# Supplementary figures and images for: Genetic diversity and structure of the critically endangered Artocarpus annulatus, a crop wild relative of jackfruit (A. heterophyllus)
Source: PeerJ. 2020 Sep 21;8:e9897. doi: 10.7717/peerj.9897 (PMC7513743; doi:10.7717/peerj.9897)

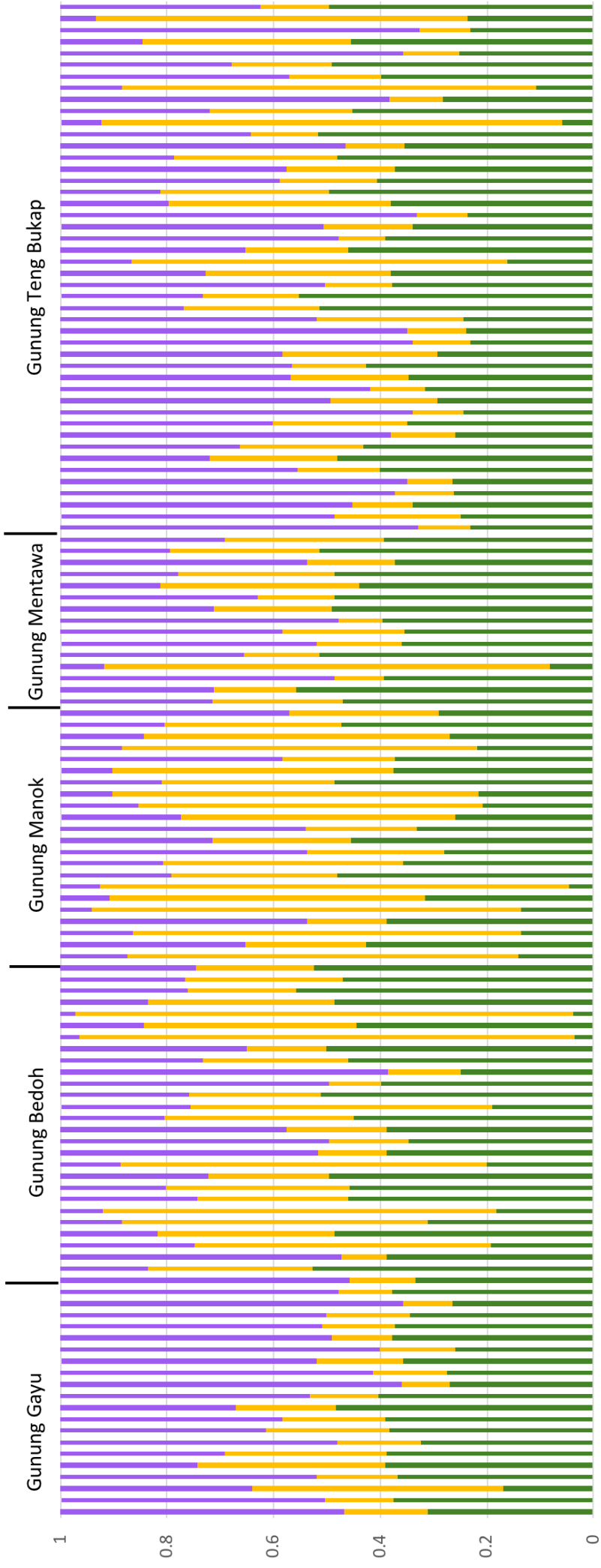

Supplement: Figure S1 — The population names are indicated above the plot. [file peerj-08-9897-s004.pdf]
